# Heat-Related Deaths — United States, 2004–2018

**DOI:** 10.15585/mmwr.mm6924a1

**Published:** 2020-06-19

**Authors:** Ambarish Vaidyanathan, Josephine Malilay, Paul Schramm, Shubhayu Saha

**Affiliations:** 1 Division of Environmental Health Science and Practice, National Center for Environmental Health, CDC.

Deaths attributable to natural heat exposure, although generally considered preventable (*1*), represent a continuing public health concern in the United States. During 2004–2018, an average of 702 heat-related deaths occurred in the United States annually. To study patterns in heat-related deaths by age group, sex, race/ethnicity, and level of urbanization, and to explore comorbid conditions associated with deaths resulting from heat exposure, CDC analyzed nationally comprehensive mortality data from the National Vital Statistics System (NVSS).* The rate of heat-related mortality tended to be higher among males, persons aged ≥65 years, non-Hispanic American Indian/Alaska Natives, and persons living in noncore nonmetropolitan and large central metropolitan counties. Natural heat exposure was a contributing cause of deaths attributed to certain chronic medical conditions and other external causes. Preparedness and response initiatives directed toward extreme heat events, currently underway at local, state, and national levels, can contribute to reducing morbidity and mortality associated with natural heat exposure. Successful public health interventions^†^ to mitigate heat-related deaths include conducting outreach to vulnerable communities to increase awareness of heat-related symptoms and provide guidance for staying cool and hydrated, particularly for susceptible groups at risk such as young athletes and persons who are older or socially isolated (*2*). Improved coordination across various health care sectors could inform local activities to protect health during periods of high heat. For instance, jurisdictions can monitor weather conditions and syndromic surveillance data to guide timing of risk communication and other measures (e.g., developing and implementing heat response plans, facilitating communication and education activities) to prevent heat-related mortality in the United States. CDC also recommends that federal, state, local, and tribal jurisdictions open cooling centers or provide access to public locations with air conditioning for persons in need of a safe, cool, environment during hot weather conditions. In light of the coronavirus disease 2019 (COVID-19) pandemic, CDC updated its guidance on the use of cooling centers to provide best practices (e.g., potential changes to staffing procedures, separate areas for persons with symptoms of COVID-19, and physical distancing) to reduce the risk for introducing and transmitting SARS COV-2, the virus that causes COVID-19, into cooling centers.^§^

Heat-related deaths among U.S. residents were identified using *International Classification of Diseases, Tenth Revision* (ICD-10)^¶^ codes included in the NVSS multiple-cause-of-death mortality data. Selected heat-related case records included those listing ICD-10 codes X30 (exposure to excessive natural heat), P81.0 (environmental hyperthermia of newborn), or T67 (effects of heat and light) as the underlying cause of death,** or as one of the contributing causes.^††^ Records with ICD-10 code W92 (exposure to excessive heat of man-made origin) listed anywhere on the death certificate were excluded to restrict the selection to deaths resulting from natural heat exposure. For case records listing heat-related codes for natural heat exposure occurring only as contributing causes, comorbid conditions recorded as the underlying cause of death were further evaluated for the following categories^§§^: major cardiovascular diseases (I00–I78) (*2*); external causes of morbidity and mortality (V01–Y98 and U01–U03); mental and behavioral disorders (F00–F99); diseases of the respiratory system (J00–J99); endocrine, nutritional and metabolic disorders (E00–E90); diseases of the digestive system (K00–K93); genitourinary disorders (N00–N98); musculoskeletal disorders (M00–M99); and other diseases. Deaths were stratified by age group, sex, race/ethnicity, and level of urbanization and combined with U.S. Census Bureau population estimates for calculating crude rates. Crude rate estimates based on fewer than 20 deaths were deemed unreliable and not reported. Analyses were performed using SAS statistical software (version 9.4; SAS Institute).

During 2004–2018, a total of 10,527 deaths resulting from exposure to heat-related conditions were identified. Approximately 90% (9,757) of these deaths occurred during May–September. The crude rate of heat-related deaths varied from year to year, with highest rates observed over the 15-year period during 2006, 2011, and 2018. Although Arizona, California, and Texas account for only approximately 23% of the U.S. population,^¶¶^ these three states accounted for approximately one third (3,852; 37%) of heat-related deaths among U.S. residents. Overall, approximately two thirds (70%) of all heat-related deaths occurred in males, and deaths among males outnumbered those among females in all age groups except infants aged <1 year (Table 1). Among the 10,470 decedents for whom age information was available, 748 (7%) were aged <15 years, 2,010 (19%) were aged 15–44 years, 3,693 (35%) were aged 45–64 years, and 4,019 (39%) were aged ≥65 years; the rate of heat-related deaths among persons aged ≥65 years was 0.7 per 100,000 population, the highest across all age groups. For the period 2004–2018, among all race/ethnicity groups, non-Hispanic whites had the highest number of heat-related deaths (6,602); however, non-Hispanic American Indian/Alaska Natives had the highest across the rate of heat-related deaths (0.6 per 100,000 population) (Table 2). Non-Hispanic blacks had the second highest number of heat-related deaths (1,965) and rate ( 0.3 per 100,000 population). Across various levels of urbanization,*** the highest heat-related mortality rates were observed among persons living in noncore nonmetropolitan (0.3 per 100,000 population) and large central metropolitan counties (0.3 per 100,000 population) (Table 2).

**TABLE 1:** Number and rate of heat-related deaths, by cause of death category,* age group, and sex — United States, 2004–2018^†^

Age group (yrs)	Cause of death category, no. (rate)^§^								
	Heat-related codes as the underlying cause			Heat-related codes as a contributing cause			All heat-related deaths		
**Total**	**Male**	**Female**	Total	Male	Female	Total	Male	Female
<1	160 (0.3)	76 (0.2)	84 (0.3)	87 (0.1)	43 (0.1)	44 (0.2)	247 (0.4)	119 (0.4)	128 (0.4)
1–4	303 (0.1)	178 (0.1)	125 (0.1)	125 (0.1)	76 (0.1)	49 (0.0)	428 (0.2)	254 (0.2)	174 (0.1)
5–14	56 (0.0)	42 (0.0)	14 (—)^¶^	17 (—)^¶^	14 (—)^¶^	3 (—)^¶^	73 (0.0)	56 (0.0)	17 (—)^¶^
15–24	234 (0.0)	203 (0.1)	31 (0.0)	94 (0.0)	77 (0.0)	17 (—)^¶^	328 (0.0)	280 (0.1)	48 (0.0)
25–34	430 (0.1)	378 (0.1)	52 (0.0)	230 (0.0)	195 (0.1)	35 (0.0)	660 (0.1)	573 (0.2)	87 (0.0)
35–44	670 (0.1)	550 (0.2)	120 (0.0)	352 (0.1)	287 (0.1)	65 (0.0)	1,022 (0.2)	837 (0.3)	185 (0.1)
45–54	1,090 (0.2)	874 (0.3)	216 (0.1)	684 (0.1)	533 (0.2)	151 (0.0)	1,774 (0.3)	1,407 (0.4)	367 (0.1)
55–64	1,024 (0.2)	762 (0.3)	262 (0.1)	895 (0.2)	658 (0.2)	237 (0.1)	1,919 (0.3)	1,420 (0.5	499 (0.2)
65–74	862 (0.2)	562 (0.3)	300 (0.2)	774 (0.2)	534 (0.3)	240 (0.1)	1,636 (0.4)	1,096 (0.7)	540 (0.3)
75–84	778 (0.4)	441 (0.5)	337 (0.3)	657 (0.3)	382 (0.4)	275 (0.2)	1,435 (0.7)	823 (1.0)	612 (0.5)
≥85	562 (0.7)	251 (0.9)	311 (0.5)	386 (0.5)	173 (0.6)	213 (0.4)	948 (1.1)	424 (1.5)	524 (0.9)
Not stated**	51 (N/A)	46 (N/A)	5 (N/A)	6 (N/A)	6 (N/A)	0 (N/A)	57 (N/A)	52 (N/A)	5 (N/A)
**All ages**	**6,220 (0.1)**	**4,363 (0.2)**	**1,857 (0.1)**	**4,307 (0.1)**	**2,978 (0.1)**	**1,329 (0.1)**	**10,527 (0.2)**	**7,341 (0.3)**	**3,186 (0.1)**

**Abbreviation:** N/A = not applicable.

* Heat-related deaths are identified using *International Classification of Diseases, Tenth Revision* cause-of-death codes X30 (exposure to excessive natural heat), P81.0 (environmental hyperthermia of newborn), and T67 (effects of heat and light) listed as the underlying cause or as one of the contributing causes in death records. Records with code W92 (exposure to excessive heat of man-made origin) listed anywhere on the death certificate were excluded from this selection.

^†^ Based on multiple-cause-of-death data from the National Center for Health Statistics (NCHS) Vital Statistics System (https://www.cdc.gov/nchs/nvss/deaths.htm) and NCHS Bridged-Race Population data (https://www.cdc.gov/nchs/nvss/bridged_race.htm). This information is available from https://wonder.cdc.gov**.**

^§^ Crude rate per 100,000 population.

^¶^ Rate estimates based on fewer than 20 deaths were deemed unreliable and not reported.

** Rate estimates were not calculated because a population denominator was unavailable.

**TABLE 2:** Number and rate of heat-related deaths,* by race/ethnicity and level of urbanization — United States, 2004–2018^†^

Characteristic	No. of deaths (rate)^§^
**Race/Ethnicity^¶^**	
Hispanic	1,349 (0.2)
American Indian/Alaska Native, non-Hispanic	241 (0.6)
Asian/Pacific Islander, non-Hispanic	194 (0.1)
Black, non-Hispanic	1,965 (0.3)
White, non-Hispanic	6,602 (0.2)
Not stated**	176 (N/A)
**Level of urbanization** ^††^	
Large central metro	4,402 (0.3)
Large fringe metro	1,607 (0.1)
Medium metro	1,764 (0.2)
Small metro	990 (0.2)
Micropolitan	879 (0.2)
Noncore	885 (0.3)
**Total**	**10,527 (0.2)**

**Abbreviation:** N/A = not applicable.

* Heat-related deaths are identified using *International Classification of Diseases, Tenth Revision* cause-of-death codes X30 (exposure to excessive natural heat), P81.0 (environmental hyperthermia of newborn), and T67 (effects of heat and light) listed as the underlying cause or as one of the contributing causes in death records. Records with code W92 (exposure to excessive heat of man-made origin) listed anywhere on the death certificate were excluded from this selection.

^†^ Based on multiple-cause-of-death data from the National Center for Health Statistics (NCHS) Vital Statistics System (https://www.cdc.gov/nchs/nvss/deaths.htm) and NCHS Bridged-Race Population data (https://www.cdc.gov/nchs/nvss/bridged_race.htm). This information is available from https://wonder.cdc.gov**.**

^§^ Crude rate per 100,000 population.

^¶^ American Indian/Alaska Native, Asian/Pacific Islander, black, and white decedents were non-Hispanic; Hispanic decedents could have been of any race.

** Rate estimates were not calculated because a population denominator was unavailable.

^††^ https://www.cdc.gov/nchs/data_access/urban_rural.htm.

Natural heat exposure–related codes were recorded as the underlying cause in 6,220 (59%) heat-related deaths, with one heat-related death attributed to environmental hyperthermia of a newborn, and the remainder from exposure to excessive natural heat (6,219; 59%) as the underlying cause. For the remaining 4,307 (41%) heat-related deaths, exposure to excessive natural heat, environmental hyperthermia of a newborn, or effects of heat and light were recorded as a contributing cause of death. When heat-related conditions were a contributing factor, as opposed to the underlying cause of death, major cardiovascular diseases (2,112; 49%) or external causes (1,543; 36%) were most often listed as the underlying cause, collectively accounting for approximately 85% of such deaths (Table 3). More specifically, natural heat exposure contributed to 1,463 (34%) deaths from ischemic heart diseases, 438 (10%) deaths from hypertension, and 773 (18%) deaths from alcohol poisoning and drug overdoses.

**TABLE 3:** Selected underlying causes* of death for which heat-related conditions were listed as a contributing factor^†^ — United States, 2004–2018^§^

Underlying cause of death^¶^	No. (%)
Major cardiovascular diseases**	**2,112 (49)**
Hypertensive diseases	438 (10)
Ischemic heart diseases	1,463 (34)
Other cardiovascular diseases	211 (5)
**External causes of morbidity and mortality** ^††^	**1,543 (36)**
Alcohol poisoning deaths	130 (3)
Drug overdose deaths	643 (15)
Other external causes of morbidity and mortality	770 (18)
**Mental and behavioral disorders** ^§§^	**174 (4)**
Mental and behavioral disorders due to psychoactive substance use	151 (4)
Other mental and behavioral disorders	23 (0)
**Diseases of the respiratory system** ^¶¶^	**127 (3)**
Chronic lower respiratory diseases	116 (3)
Other diseases of the respiratory system	11 (0)
**Endocrine, nutritional, and metabolic disorders** ***	**128 (3)**
Diabetes mellitus	78 (2)
Other endocrine, nutritional, and metabolic disorders	50 (1)
**Diseases of the digestive system** ^†††^	**48 (1)**
Diseases of the liver	33 (1)
Other diseases of the digestive system	15 (0)
**Genitourinary disorders** ^§§§^	**30 (1)**
**Musculoskeletal disorders** ^¶¶¶^	**12 (0)**
**Other diseases**	**133 (3)**
Total underlying causes of death with heat-related conditions**** **as a contributing factor**	**4,307 (100)**

* The underlying cause of death was defined as the disease or injury that initiated the chain of events that led directly and inevitably to death. https://icd.who.int/browse10/Content/statichtml/ICD10Volume2_en_2010.pdf.

^†^ Contributing conditions, or factors, were defined as diseases, injuries, or complications that contributed to the death and were a result of the underlying cause. https://icd.who.int/browse10/Content/statichtml/ICD10Volume2_en_2010.pdf.

^§^ Based on multiple-cause-of-death data from the National Center for Health Statistics Vital Statistics System (https://www.cdc.gov/nchs/nvss/deaths.htm). This information is available from https://wonder.cdc.gov.

^¶^ Deaths were classified using *International Classification of Diseases, Tenth Revision* codes.

** Major cardiovascular diseases were identified using underlying cause-of-death codes I00–I78. Hypertensive diseases and ischemic heart diseases were identified using underlying cause-of-death codes I10–I15 and I20–I25.

^††^ External causes of mortality were identified using underlying cause-of-death codes V01–Y98 and U01–U03. Alcohol poisoning deaths were identified using underlying cause-of-death codes X45, X65, and Y15. Drug overdose deaths were identified using underlying cause-of-death codes X40–X44, X60–X64, X85, and Y10–Y14.

^§§^ Mental and behavioral disorders were identified using underlying cause-of-death codes F00–F99. Mental and behavioral disorders attributed to psychoactive substance use were identified using underlying cause-of-death codes F10–F19.

^¶¶^ Diseases of the respiratory system were identified using underlying cause-of-death codes J00–J99. Chronic lower respiratory diseases were identified using underlying cause-of-death codes J40–J47.

*** Endocrine, nutritional and metabolic disorders were identified using underlying cause-of-death codes E00–E90. Diabetes mellitus diseases were identified using underlying cause-of-death codes E10–E14.

^†††^ Diseases of the digestive system were identified using underlying cause-of-death codes K00–K93. Diseases of the liver were identified using underlying cause-of-death codes K70–K77.

^§§§^ Genitourinary disorders were identified using underlying cause-of-death codes N00–N98.

^¶¶¶^ Musculoskeletal disorders were identified using underlying cause-of-death codes M00–M99.

**** Heat-related conditions were identified using cause-of-death codes X30 (exposure to excessive natural heat), P81.0 (environmental hyperthermia of newborn), and T67 (effects of heat and light). Records with code W92 (exposure to excessive heat of man-made origin) listed anywhere on the death certificate were excluded from this selection.

## Discussion

Heterogeneity in population-level vulnerability to extreme heat events is evident and is distributed differentially across and within communities (*3*). Social determinants of health^†††^ and access to health care vary with levels of urbanization and play a role in determining resiliency of communities to extreme heat events and other disasters. Observed differences in heat-related mortality across racial/ethnic groups can also be associated with social vulnerability, which often tracks with factors leading to heat exposure (e.g., less green space and more heat-absorbing surfaces), health disparities manifested by lower income, and absence of structural adaptations such as air conditioning (*3*). In addition, persons at risk, including those who are older, might have higher susceptibility to heat stress because of impaired thermoregulatory responses, social isolation, or both (*4*); in this analysis, persons aged ≥65 years accounted for approximately 40% of all heat-related deaths and experienced the highest rate of heat-related deaths among all age groups. However, even young and healthy persons are at risk for heat stress when engaging in strenuous outdoor physical activities during hot weather (*5*). Similarly, sex differences in thermoregulatory response and participation in high-risk outdoor occupations, such as working in agriculture and the construction industry, might explain the higher heat-related mortality observed in males (*6*,*7*).

Past studies have demonstrated a relationship between ambient temperatures and mortality (*8*). In particular, extreme heat exposure can exacerbate certain chronic medical conditions, including hypertension and heart disease (*4*,*5*). In addition, medications that are typically used to treat these chronic medical conditions such as beta-blockers, diuretics, and calcium-channel blockers, can interfere with thermoregulation and result in a reduced ability to respond to heat stress (*5*). Further, use of alcohol or drugs (e.g., methamphetamine and cocaine.) are known risk factors for heat-related deaths (*5*); in this analysis, exposure to environmental heat was a contributing cause for several alcohol poisoning and drug overdose deaths. A significant increase in mortality risk associated with unintentional cocaine overdose has been reported during periods of extreme heat (*9*). Of note, death records with mental and behavioral disorders as the underlying cause of death had heat-related ICD-10 codes as contributing causes. In addition to compromising the body’s ability to cope with heat stress, certain psychiatric conditions can alter risk perception and reduce awareness to prevailing hot conditions (*4*,*10*).

The findings in this report are subject to at least three limitations. First, heat exposure is a contributing factor to deaths resulting from many causes. Data collected by NVSS might not capture the full spectrum of heat stress–related deaths, especially if excessive heat is not explicitly documented in death records. In addition, heat-related deaths among non-U.S. residents are not presented here because a reliable population denominator is not available for calculating crude rates. Second, persons might be exposed to environmental heat at multiple locations, but deaths reported here are attributed to decedents’ place of residence. Finally, detailed NVSS data describing the circumstances of death might be limited and vary on the basis of information available from the medical certification of death process, resources, and the person completing the death certificate. Therefore, in some instances, these data might not provide the necessary information to explain the situation or series of events leading to death from excessive heat exposure (*1*).

Understanding patterns in heat-related mortality associated with comorbidity, age group, sex, race/ethnicity, and urbanization levels could assist CDC and its public health partners in developing more effective surveillance and intervention strategies that integrate environmental health and other public health domains. Implementing these interventions during the COVID-19 pandemic might require additional considerations; for example, staff members of cooling centers need to be able to mitigate risk for transmission of SARS COV-2 among visitors and staff members. Measures to prevent heat-related mortality can include conducting routine and near-real-time surveillance (e.g. syndromic surveillance), developing and implementing heat response plans, facilitating communication and education activities, and operating cooling centers. A coordinated approach across health care sectors is needed to prevent heat-related mortality in the United States.

**Summary**

**What is already known about this topic?**

Deaths attributed to natural heat exposure represent a continuing public health concern. Preparedness and response initiatives that limit exposure during periods of extreme heat can reduce mortality.

**What is added by this report?**

During 2004–2018, an average of 702 heat-related deaths (415 with heat as the underlying cause and 287 as a contributing cause) occurred in the United States annually. Natural heat exposure was a contributing cause of death attributed to certain chronic medical conditions, alcohol poisoning, and drug overdoses.

**What are the implications for public health practice?**

A coordinated approach across health care sectors to prevent heat-related mortality can include conducting syndromic surveillance, developing and implementing heat response plans, facilitating communication and education activities, and operating cooling centers.

## Footnotes

* https://www.cdc.gov/nchs/nvss/index.htm.

† https://www.cdc.gov/disasters/extremeheat/index.html.

§ https://www.cdc.gov/coronavirus/2019-ncov/php/cooling-center.html.

¶ https://www.cdc.gov/nchs/icd/icd10.htm.

** The underlying cause of death was defined as the disease or injury that initiated the chain of events that led directly and inevitably to death. https://icd.who.int/browse10/Content/statichtml/ICD10Volume2_en_2010.pdf.

†† Contributing conditions, or factors, were defined as diseases, injuries, or complications that contributed to the death and were a result of the underlying cause. https://icd.who.int/browse10/Content/statichtml/ICD10Volume2_en_2010.pdf.

§§
https://www.cdc.gov/nchs/data/dvs/2e_volume1_2017.pdf .

¶¶ https://www.census.gov/2010census/data/.

*** https://www.cdc.gov/nchs/data/series/sr_02/sr02_166.pdf.

††† https://svi.cdc.gov/.
